# Author's reply

**DOI:** 10.4103/0972-2327.61289

**Published:** 2010

**Authors:** Ambar Chakravarty

**Affiliations:** Department of Neurology, Vivekananda Institute of Medical Sciences, Kolkata, India E-mail: saschakra@yahoo.com

Sir,

I thank the author of the letter for his/her comments regarding my article. Basically, the letter stresses the same issue as highlighted in my article namely stressing on finding of hidden talents in cognitively challenged children rather than putting undue pressure on pursuing conventional educational training. That had been the message from the film as well. For further information on savants and creativity, I would refer to two articles written by me recently.[1,2]
